# Comparison of surgical and oncologic outcomes in very elderly patients (≥ 80 years old) and elderly (65–79 years old) colorectal cancer patients: a propensity score matching

**DOI:** 10.1186/s12876-022-02277-y

**Published:** 2022-04-25

**Authors:** Yu-Xi Cheng, Xiao-Yu Liu, Bing Kang, Wei Tao, Zheng-Qiang Wei, Dong Peng

**Affiliations:** 1 Department of Gastrointestinal Surgery, The First Affiliated Hospital of Chongqing, Medical University, Chongqing, 400016 China; 2 Department of Clinical Nutrition, The First Affiliated Hospital of Chongqing Medical University, Chongqing, 400016 China

**Keywords:** Colorectal cancer, Elderly, Overall survival, Disease-free survival, Propensity score matching

## Abstract

**Purpose:**

The purpose of this study was to investigate the short-term outcomes and prognosis of elderly and very elderly colorectal cancer (CRC) patients after primary CRC surgery using propensity score matching (PSM).

**Methods:**

This study retrospectively collected the medical records of CRC patients ≥ 65 years old undergoing primary CRC surgery from Jan 2011 to Jan 2020. Short-term outcomes, overall survival (OS) and disease-free survival (DFS) were compared between very elderly CRC patients (≥ 80 years old) and elderly CRC patients (65–79 years old).

**Results:**

A total of 2084 patients were enrolled for analysis. After PSM, 331 very elderly patients were matched to 331 elderly patients. In terms of short-term outcomes, the very elderly patients had longer postoperative hospital stays (p = 0.007) after PSM. In terms of OS, it was found that age (p < 0.01, HR = 1.878, 95% CI 1.488–2.371), tumor stage (p < 0.01, HR = 1.865, 95% CI 1.603–2.170), overall complications (p < 0.01, HR = 1.514, 95% CI 1.224–1.872) and major complications (p = 0.001, HR = 2.012, 95% CI 1.319–3.069) were independent prognostic factors. For DFS, age (p < 0.01, HR = 1.816, 95% CI 1.579–2.088), tumor stage (p < 0.01, HR = 1.816, 95% CI 1.579–2.088), overall complications (p = 0.002, HR = 1.379, 95% CI 1.128–1.685) and major complications (p = 0.002, HR = 1.902, 95% CI 1.259–2.874) were found to be independent prognostic factors. Moreover, elderly patients had a better OS and DFS than very elderly patients.

**Conclusion:**

Very elderly patients had a poorer prognosis than elderly patients after primary CRC surgery. Surgeons should be cautious when treating very elderly CRC patients.

## Background

Colorectal cancer (CRC) is the fourth most commonly occurring cancer and the third-leading cause of cancer-related death globally [1]. The number of newly diagnosed cases of CRC reached 2 million, and cancer-related deaths were expected to reach 1 million in 2018 [1]. Radical CRC surgery is the primary course of treatment in resectable cases [2]. Predictive risk factors for the occurrence of CRC include age, alcohol consumption, a high-fat diet and physical inactivity [3–5].

It was reported that 16% of the world population would be ≥ 65 years of age by 2050 due to the aging of society, with an expected increase in elderly patients with CRC [6]. More elderly patients underwent CRC surgery and received chemoradiotherapy after surgery because of the updated techniques with higher safety and effectiveness [7]. However, compared with nonelderly patients, elderly patients usually had more comorbidities, such as type 2 diabetes (T2DM), cardiopulmonary insufficiency or chronic renal insufficiency.

In previous studies, age was an independent risk factor for in-hospital complications and mortality after CRC surgery [8–10]. Patients ≥ 80 years of age can potentially suffer from severe complications and mortality [11]. However, no previous studies have reported comparisons between elderly and very elderly CRC patients. Therefore, the purpose of this study was to investigate the short-term outcomes and prognosis of elderly and very elderly CRC patients after primary CRC surgery.

## Methods

### Patients

We retrospectively collected the medical records of CRC patients undergoing primary CRC surgery from Jan 2011 to Jan 2020 in a clinical center. This study was conducted in accordance with the World Medical Association Declaration of Helsinki. Ethical approval was obtained from the Institutional Ethics Committee of the local hospital (2021-517).

### Inclusion and exclusion criteria

Patients who underwent primary CRC surgery and were diagnosed pathologically with CRC after surgery were initially included in this study (n = 5473). The exclusion criteria were as follows: 1, pathologically diagnosed stage IV CRC (n = 875); 2, an age of < 65 years (n = 2166); 3, incomplete medical records (n = 323); and 4, non-R0 resection (n = 25). Therefore, a total of 2084 patients were enrolled in the final analysis.

### Peri-operative management and surveillance

Radical colorectal resection and lymph node dissection were routinely performed in all patients according to the AJCC 8th Edition [12].

All patients who underwent CRC surgery were advised to receive regular laboratory evaluation and appropriate exercise for postoperative recovery. Follow-up was recommended every three months after CRC surgery for the first three years and every six months for the next two years. The follow-up included carcinoembryonic antigen (CEA) testing, computed tomography (CT), magnetic resonance imaging (MRI) and colonoscopy.

### Definitions

The patients were divided into the following two groups: elderly patients were defined from 65 to 79 years old, and very elderly patients were defined as ≥ 80 years of age. The tumor node metastasis (TNM) stage was documented in accordance with the AJCC 8^th^ Edition [12]. R0 resection was defined as a negative margin on pathological examination. Postoperative complications were graded by the Clavien-Dindo classification, [13] and the major complications were defined as ≥ grade III, which required surgery, endoscopy or radiological intervention. Overall survival (OS) was calculated from CRC surgery to death or last follow-up. Disease-free survival (DFS) was calculated from CRC surgery to recurrence, metastasis, death or last follow-up.

### Data collection

The baseline information and short-term outcomes were collected from electronic medical records for analysis. The baseline information included age, sex, body mass index (BMI), T2DM, smoking, drinking, hypertension, coronary heart disease (CHD), surgical methods, tumor location and tumor stage. The short-term outcomes included operation time, blood loss, retrieved lymph nodes, postoperative hospital stay, overall complications and major complications. The follow-up results were collected from the outpatient department records or through telephone interviews.

### Propensity score matching (PSM)

To minimize the selection bias in baseline characteristics between the two groups, [14] very elderly patients were matched to elderly patients using the PSM method in this study. Nearest neighbor matching was performed without replacement at a 1:1 ratio, and a caliper width with a 0.01 standard deviation (SD) was specified.

### Statistical analysis

Continuous variables are expressed as the mean ± SD, and an independent-sample t test was used to compare the difference between the elderly and very elderly groups. Categorical variables are expressed as n (%), and the chi-square test or Fisher's exact test was used. Univariate and multivariate logistic regression analyses were used to identify predictors of overall complications. Predictive factors for OS and DFS were identified through Cox regression analyses. The Kaplan–Meier method was used to compare OS and DFS between the elderly and very elderly groups. Furthermore, multivariate linear regression was conducted between the length of postoperative hospital stay and the patient’s clinical characteristics. Data were analyzed using SPSS (version 22.0) statistical software. A result was considered statistically significant when the bilateral p value was < 0.05.

## Results

### Patients

**Fig. 1:**
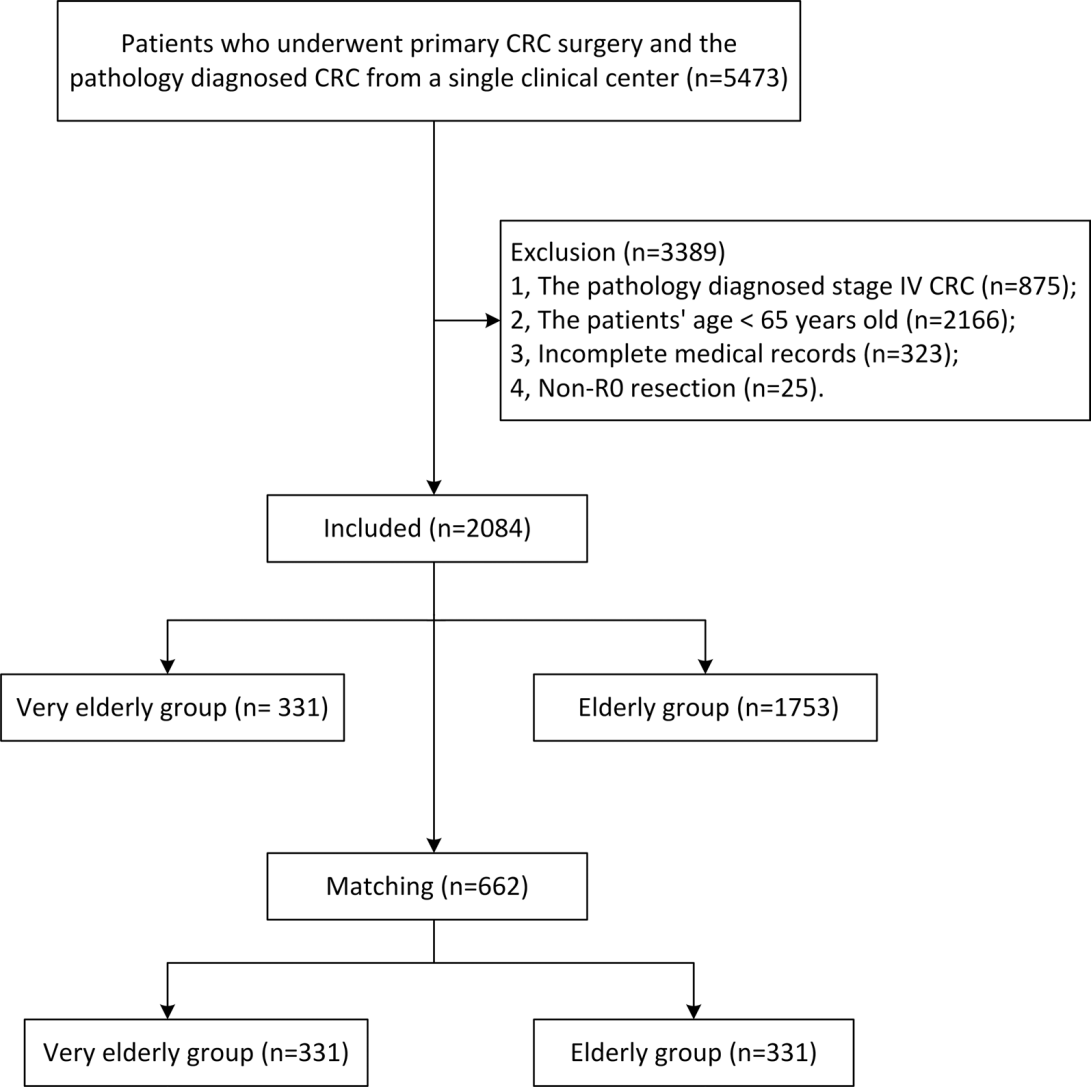
Flowchart of study selection. *Note:* CRC: colorectal cancer

A total of 5473 patients were identified in a single clinical database. After adjusting for the inclusion and exclusion criteria, there were 2084 patients enrolled for analysis, including 331 very elderly patients and 1753 elderly patients. Then, after using a 1:1 ratio for PSM, 331 very elderly patients were matched to 331 elderly patients. The flow chart is shown in Fig. 1.

### Baseline characteristics

Very elderly patients and elderly patients were compared in terms of the baseline characteristics. Elderly patients had a younger age (p < 0.01), a higher BMI (p = 0.017), a higher proportion of smoking (p < 0.01), a higher proportion of drinking (p < 0.01), a lower proportion of hypertension (p < 0.01) and a lower proportion of CHD (p < 0.01). Moreover, tumor location (p = 0.001) and tumor stage (p = 0.035) were significantly different. No significant difference was found after PSM except for age (Table 1).

**Table 1:** Baseline characteristics before and after PSM

Characteristics	Before PSM			After PSM		
	Very elderly (331)	Elderly (1753)	P value	Very elderly (331)	Elderly (331)	P value
Age (year)	83.4 ± 3.0	71.1 ± 4.1	< 0.01**	83.4 ± 3.0	71.8 ± 4.3	< 0.01**
Sex			0.104			0.436
Male	183 (55.3%)	1053 (60.1%)		183 (55.3%)	173 (52.3%)	
Female	148 (44.7%)	700 (39.9%)		148 (44.7%)	158 (47.7%)	
BMI (kg/m^2^)	22.0 ± 3.4	22.5 ± 3.3	0.017*	22.0 ± 3.4	21.8 ± 3.1	0.559
T2DM	67 (20.2%)	289 (16.5%)	0.096	67 (20.2%)	62 (18.7%)	0.624
Smoking	94 (28.4%)	675 (38.5%)	< 0.01**	96 (28.4%)	95 (28.7%)	0.864
Drinking	65 (19.6%)	547 (31.2%)	< 0.01**	65 (19.6%)	74 (22.4%)	0.390
Hypertension	158 (47.7%)	591 (33.7%)	< 0.01**	158 (47.7%)	162 (48.9%)	0.756
CHD	42 (12.7%)	115 (6.7%)	< 0.01**	42 (12.7%)	34 (10.3%)	0.329
Surgical methods			0.428			0.844
Open	63 (19.0%)	302 (17.2%)		63 (19.0%)	65 (19.6%)	
Laparoscopic	268 (81.0%)	1451 (82.8%)		268 (81.0%)	266 (80.4%)	
Tumor location			0.001**			0.383
Colon	192 (58.0%)	838 (47.8%)		192 (58.0%)	203 (61.3%)	
Rectum	139 (42.0%)	915 (52.2%)		139 (42.0%)	128 (38.7%)	
Tumor stage			0.035*			0.288
I	53 (16.0%)	321 (18.3%)		53 (16.0%)	49 (14.8%)	
II	134 (40.5%)	801 (45.7%)		134 (40.5%)	154 (46.5%)	
III	144 (43.5%)	631 (36.0%)		144 (43.5%)	128 (38.7%)	

T2DM, type 2 diabetes mellitus; BMI, body mass index; PSM, propensity score matching; CHD, coronary heart disease

Variables are expressed as the mean ± SD, n (%), *P-value < 0.05, ** P-value < 0.01

### Short-term outcomes

Short-term outcomes were compared between the two groups, including operation time, blood loss, retrieved lymph nodes, postoperative hospital stay, overall complications and major complications. Very elderly patients had longer postoperative hospital stays (p = 0.015) and higher overall complications (p < 0.01) than elderly patients. After PSM, very elderly patients also had longer postoperative hospital stays (p = 0.007) (Table 2).

**Table 2:** Short-term outcomes before and after PSM

Characteristics	Before PSM			After PSM		
	Very Elderly (331)	Elderly (1753)	P value	Very Elderly (331)	Elderly (331)	P value
Operation time (min)	215.2 ± 69.2	222.7 ± 82.1	0.118	215.2 ± 69.2	211.4 ± 75.8	0.507
Blood loss (mL)	108.9 ± 131.2	102.1 ± 134.7	0.399	108.9 ± 131.2	101.3 ± 148.9	0.484
Retrieved lymph nodes	14.3 ± 8.4	14.3 ± 7.0	0.935	14.3 ± 8.4	14.8 ± 6.9	0.415
Hospital stay (day)	13.4 ± 10.9	11.9 ± 10.6	0.015*	13.4 ± 10.9	11.5 ± 6.7	0.007**
Overall complications	118 (35.6%)	438 (25.0%)	< 0.01**	118 (35.6%)	97 (29.3%)	0.081
Major complications	15 (4.5%)	51 (2.9%)	0.122	15 (4.5%)	11 (3.3%)	0.424

T2DM, type 2 diabetes mellitus; PSM, propensity score matching

Variables are expressed as the mean ± SD, n (%), *P-value < 0.05, ** P-value < 0.01

### Univariate and multivariate analysis of OS

The median follow-up time was 37 (1–114) months. To identify predictive risk factors for OS, we carried out the univariate and multivariate analyses. In univariate analysis, major complications (p < 0.01, HR = 2.173, 95% CI 1.460–3.235), overall complications (p < 0.01, HR = 1.638, 95% CI 1.340–2.002), tumor stage (p < 0.01, HR = 1.826, 95% CI 1.571–2.123), tumor location (p = 0.020, HR = 1.262, 95% CI 1.037–1.536) and age (p < 0.01, HR = 1.967, 95% CI 1.561–2.478) were considered as predictors. Furthermore, in multivariate analysis, we found that four independent prognostic factors for OS, which were as follows: age (p < 0.01, HR = 1.878, 95% CI 1.488–2.371), tumor stage (p < 0.01, HR = 1.865, 95% CI 1.603–2.170), overall complications (p < 0.01, HR = 1.514, 95% CI 1.224–1.872) and major complications (p = 0.001, HR = 2.012, 95% CI 1.319–3.069) (Table 3).

**Table 3:** Univariate and multivariate analysis of overall survival

Risk factors	Univariate analysis		Multivariate analysis		
	HR (95% CI)	P value	HR (95% CI)		P value
Age (very elderly/elderly)	1.967 (1.561–2.478)	< 0.01**	1.881 (1.490–2.375)		< 0.01**
Sex (male/female)	0.876 (0.717–1.071)	0.197			
BMI (>/≤ 22.3)	0.837 (0.687–1.020)	0.078	0.890 (0.730–1.085)		0.249
Hypertension (yes/no)	0.890 (0.723–1.096)	0.273			
T2DM (yes/no)	1.048 (0.803–1.367)	0.730			
Tumor location (colon/ rectum)	1.262 (1.037–1.536)	0.020*	1.192 (0.977–1.453)		0.083
Tumor stage (III/II/I)	1.826 (1.571–2.123)	< 0.01**	1.856 (1.595–2.160)		< 0.01**
Smoking (yes/no)	1.128 (0.923–1.378)	0.241			
Drinking (yes/no)	1.035 (0.835–1.284)	0.752			
CHD (yes/no)	1.065 (0.725–1.564)	0.748			
Overall complications (yes/no)	1.638 (1.340–2.002)	< 0.01**	1.513 (1.224–1.871)		< 0.01**
Major complications (yes/no)	2.173 (1.460–3.235)	< 0.01**	2.022 (1.325–3.084)		0.001**

HR, Hazard ratio; CI, confidence interval; BMI, body mass index; T2DM, type 2 diabetes mellitus; CHD, coronary heart disease

* P-value < 0.05, ** P-value < 0.01

### Univariate and multivariate analysis of DFS

In terms of DFS, we found four predictive risk factors in univariate analysis, including major complications (p = 0.001, HR = 1.992, 95% CI 1.350–2.939), overall complications (p < 0.01, HR = 1.500, 95% CI 1.240–1.813), tumor stage (p < 0.01, HR = 1.788, 95% CI 1.554–2.056) and age (p < 0.01, HR = 1.826, 95% CI 1.467–2.274). In multivariate analysis, four independent prognostic factors were found for DFS, which included as follows: age (p < 0.01, HR = 1.816, 95% CI 1.579–2.088), tumor stage (p < 0.01, HR = 1.816, 95% CI = 1.579–2.088), overall complications (p = 0.002, HR = 1.379, 95% CI 1.128–1.685), and major complications (p = 0.002, HR = 1.902, 95% CI 1.259–2.874) (Table 4).

**Table 4:** Univariate and multivariate analysis of disease-free survival

Risk factors	Univariate analysis		Multivariate analysis		
	HR (95% CI)	P value	HR (95% CI)		P value
Age (very elderly/elderly)	1.826 (1.467–2.274)	< 0.01**	1.816 (1.579–2.088)		< 0.01**
Sex (male/female)	0.879 (0.729–1.060)	0.178			
BMI (> / ≤ 22.6)	0.888 (0.739–1.068)	0.207			
Hypertension (yes/no)	0.885 (0.728–1.075)	0.217			
T2DM (yes/no)	0.963 (0.747–1.240)	0.769			
Tumor site (colon/ rectum)	1.165 (0.970–1.399)	0.103			
Tumor stage (I/II/III)	1.788 (1.554–2.056)	< 0.01**	1.816 (1.579–2.088)		< 0.01**
Smoking (yes/no)	1.114 (0.923–1.343)	0.261			
Drinking (yes/no)	1.019 (0.833–1.247)	0.856			
CHD (yes/no)	1.068 (0.749–1.522)	0.716			
Overall complications (yes/no)	1.500 (1.240–1.813)	< 0.01**	1.379 (1.128–1.685)		0.002**
Major complications (yes/no)	1.992 (1.350–2.939)	0.001**	1.902 (1.259–2.874)		0.002**

HR, Hazard ratio; CI, confidence interval; BMI, body mass index; T2DM, type 2 diabetes mellitus; CHD, coronary heart disease

* P-value < 0.05, ** P-value < 0.01

### Prognosis before and after PSM

**Fig. 2:**
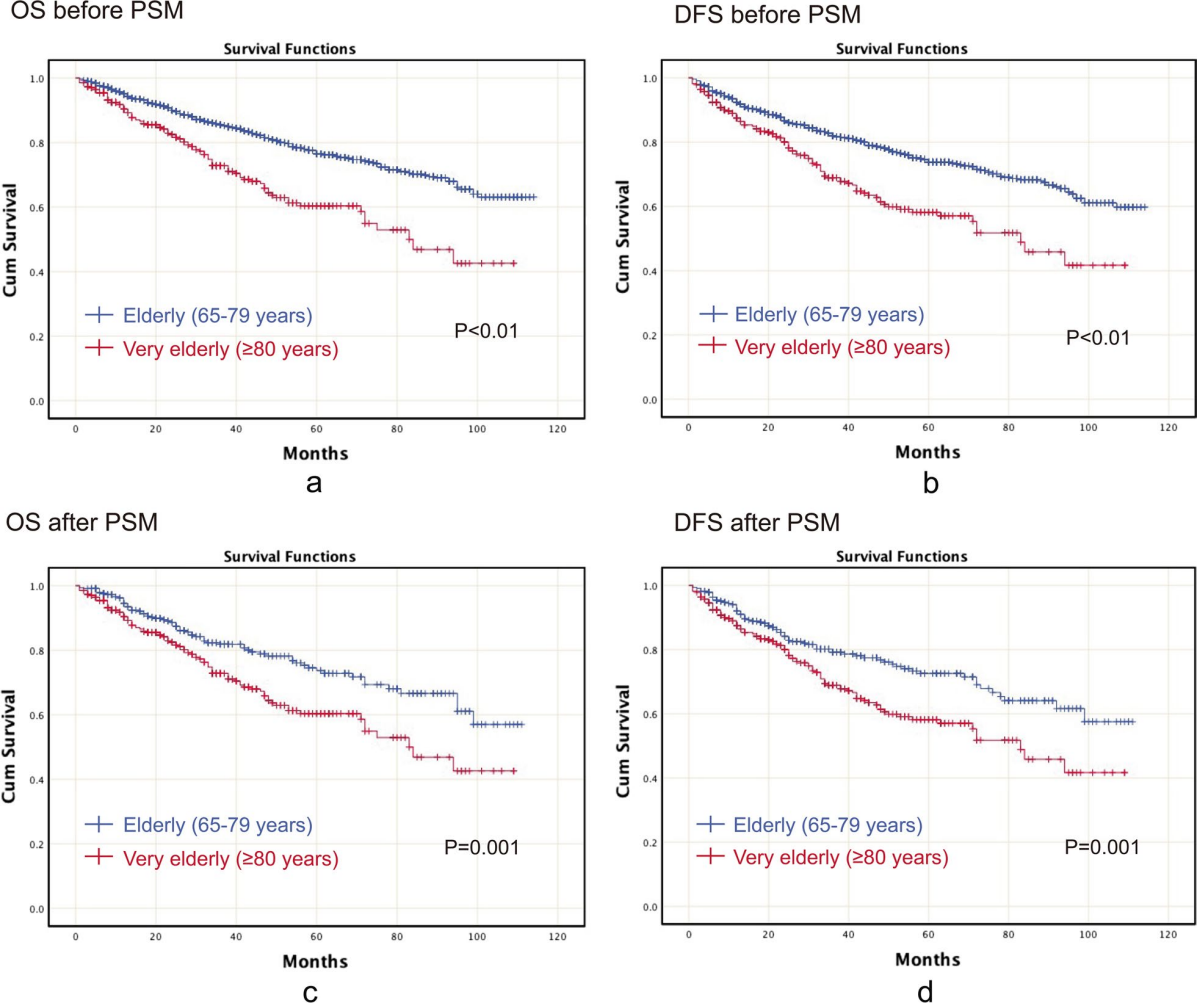
The Kaplan–Meier curve before and after PSM for OS and DFS. **a** OS before PSM; **b** DFS before PSM; **c** OS after PSM; **d** DFS after PSM. *Note:* PSM, propensity score matching; OS, overall survival; DFS, disease-free survival

Kaplan–Meier curves were generated before and after PSM for OS and DFS, respectively. Before PSM, elderly patients had better OS (p < 0.01) and better DFS (p < 0.01) than very elderly patients (Fig. 2a, b). Furthermore, after PSM, elderly patients had better OS (p = 0.001) and better DFS (p = 0.001) than very elderly patients (Fig. 2c, d).

### Multivariate analysis of the very elderly patients

To explore the predictive factors of overall complications and postoperative hospital stay, we conducted multivariate analysis in very elderly patients. Regarding overall complications, open CRC surgery (p = 0.002, OR = 2.552, 95% CI = 1.410–4.618) was an independent predictor (Table 5). In terms of postoperative hospital stay, operation time (β = 0.114, p = 0.046) was significantly correlated with the postoperative hospital stay (Table 6).

**Table 5:** Univariate and multivariate analysis of the overall complications for the very elderly patients

Risk factors	Univariate analysis		Multivariate analysis	
	OR (95% CI)	P value	OR (95% CI)	P value
Surgical methods (open/laparoscopic)	2.568 (1.469–4.490)	0.001**	2.552 (1.410–4.618)	0.002**
Sex (male/female)	0.863 (0.548–1.358)	0.524		
BMI, kg/m^2^	0.947 (0.884–1.013)	0.112		
Hypertension (yes/no)	0.839 (0.534–1.317)	0.445		
T2DM (yes/no)	1.094 (0.628–1.908)	0.750		
Tumor location (colon/ rectum)	0.876 (0.556–1.381)	0.569		
Tumor stage (III/II/I)	0.917 (0.672–1.251)	0.584		
Smoking (yes/no)	1.332 (0.814–2.178)	0.254		
Drinking (yes/no)	1.163 (0.664–2.035)	0.598		
CHD (yes/no)	1.266 (0.653–2.454)	0.485		
Operation time, min	1.003 (1.000–1.006)	0.069	1.003 (0.999–1.006)	0.155
Blood loss, mL	1.002 (1.000–1.003)	0.060	1.000 (0.999–1.002)	0.676

OR, Odds ratio; CI, confidence interval; BMI, body mass index; T2DM, type 2 diabetes mellitus; CHD, coronary heart disease

* P-value < 0.05, ** P-value < 0.01

**Table 6:** Multivariate linear regression between the post-operative hospital stay and the clinical characteristics

Risk factors	β	P value
Operation time, min	0.114	0.046*
BMI, kg/m^2^	− 0.030	0.589
Blood loss, mL	0.104	0.077
Retrieved lymph nodes	− 0.087	0.116

BMI, body mass index

^*^P-value < 0.05

## Discussion

A total of 2084 patients were included in this study. To balance the difference in baseline characteristics, 331 very elderly patients were matched to 331 elderly patients using the PSM method. In terms of short-term outcomes, very elderly patients had longer postoperative hospital stays than elderly patients. Elderly patients had better OS and DFS than very elderly patients. In multivariate analysis, it was found that age, tumor stage, overall complications and major complications were independent prognostic factors for OS and DFS.

It was reported that age was associated with prognosis in terms of treating malignant tumors [15, 16]. In general, elderly patients were more likely to have cardio-cerebrovascular and pulmonary comorbidities, which affected the postoperative recovery and the sequential postoperative chemoradiotherapy [17, 18]. Utsumi M et al. [19] compared the surgical outcomes between the elderly group (≥ 80 years old) and the nonelderly group (< 80 years old) using the PSM method and found that age was not a predictor of postoperative complications after CRC surgery; however, the conclusion was unconvincing due to limited data. Thus, we further investigated whether age (focused on aged CRC patients) had an effect on short-term outcomes or prognosis after CRC surgery using the PSM method.

PSM was a relatively optimal method to minimize the bias of baseline information, and after matching, no significant difference was found in the elderly and very elderly groups. The cutoff age varied when defining the elderly and the very elderly groups [9, 10]. In this study, we chose 80 years of age as the cutoff age for analysis, which was consistent with the majority of previous studies [19–22].

As previous studies reported, postoperative complications were associated with longer postoperative hospital stays, heavier financial burdens, lower quality of life and worse prognoses [23, 24]. Odermatt M et al. [25] reported that elderly CRC patients experienced more major complications after surgery, including cardio-cerebrovascular accidents, pulmonary infections, deep venous thrombosis and anastomotic leakage. Furthermore, it was reported that age was an independent risk factor for anastomotic leakage [26]. However, we analyzed the current data and found no significant difference in postoperative complications between elderly and very elderly patients. The reason might be that the baseline information was matched between the two groups. In addition, very elderly patients had longer postoperative hospital stays than elderly patients in this study. Potential malnutrition and poor healing ability might contribute to this result. Furthermore, for very elderly patients, open CRC surgery was an independent predictor of overall complications, and operation time was significantly correlated with the postoperative hospital stay. Therefore, laparoscopic surgery with a shorter operation time is recommended for very elderly CRC patients [27, 28].

Most studies reported that CRC patients (age ≥ 80 years old) had higher mortality after CRC surgery [29, 30]. Chan et al. [31] reported that 36.8% of elderly patients suffered from pneumonia and respiratory failure, the leading cause of mortality after CRC surgery. Hinoi et al. [20] reported that the 3-year OS rate in elderly (age ≥ 80 years old) colon cancer patients with stage I-III disease was 85.5%, and the 3-year OS rate was 78.6% in elderly (age ≥ 80 years old) rectal cancer patients. In this study, we found that elderly patients had better OS than very elderly patients. Furthermore, tumor stage and complications were predictors of OS. Aging leads to a progressive decline in the functional reserve of multiple organ systems; [32] therefore, very elderly patients have worse OS.

In fact, the treatment for elderly CRC patients is based on the assessment of physiological age, patient life expectancy, and tolerance to treatment [33]. Very elderly patients who commonly have concurrent impaired liver or renal function might be undertreated for malignancy, which leads to the rapid recurrence of tumors and worse DFS [32]. In our study, very elderly patients had worse DFS than elderly patients, which could be explained by the undertreatment of very elderly patients.

Interestingly, the process of aging is highly individualized, and discrepancies between physiological and chronological age are a challenge for surgeons [25, 35]. Therefore, the surgical strategy and postoperative chemotherapy regimen should be conducted individually.

To our knowledge, this was the first study to compare the short-term outcomes and prognosis between very elderly patients and elderly patients using the PSM method. However, there were some limitations in this study. First, this was a single-center retrospective study, which might cause bias. Second, the median follow-up time was relatively short. Thus, multicenter prospective randomized controlled trials with comprehensive perioperative information should be performed in the future.

In conclusion, very elderly patients had a poorer prognosis than elderly patients after primary CRC surgery. Surgeons should be cautious about aged CRC patients.

## Data Availability

The datasets generated and/or analysed during the current study are not publicly available due [The database from our clinical center were relatively private] but are available from the corresponding author on reasonable request.

## Abbreviations

CRC: Colorectal cancer
PSM: Propensity score matching
OS: Overall survival
DFS: Disease-free survival
T2DM: Type 2 diabetes
CEA: Carcinoembryonic antigen
CT: Computed tomography
MRI: Magnetic resonance imaging
TNM: Tumor node metastasis
BMI: Body mass index
CHD: Coronary heart disease
SD: Standard deviation
